# Health inequalities in Ethiopia: modeling inequalities in length of life within and between population groups

**DOI:** 10.1186/1475-9276-12-52

**Published:** 2013-07-11

**Authors:** Eirik Joakim Tranvåg, Merima Ali, Ole Frithjof Norheim

**Affiliations:** 1 Department of Global Public Health and Primary Care, University of Bergen, N-5020, Bergen, Norway; 2 Chr. Michelsen Institute, N-5892, Bergen, Norway

**Keywords:** Ethiopia, Justice, Health inequality, Inequality in length of life, Priority setting, Inequality measurement, Gini

## Abstract

**Background and objectives:**

Most studies on health inequalities use average measures, but describing the distribution of health can also provide valuable knowledge. In this paper, we estimate and compare within-group and between-group inequalities in length of life for population groups in Ethiopia in 2000 and 2011.

**Methods:**

We used data from the 2011 and 2000 Ethiopia Demographic and Health Survey and the Global Burden of Disease study 2010, and the MODMATCH modified logit life table system developed by the World Health Organization to model mortality rates, life expectancy, and length of life for Ethiopian population groups stratified by wealth quintiles, gender and residence. We then estimated and compared within-group and between-group inequality in length of life using the Gini index and absolute length of life inequality.

**Results:**

Length of life inequality has decreased and life expectancy has increased for all population groups between 2000 and 2011. Length of life inequality within wealth quintiles is about three times larger than the between-group inequality of 9 years. Total length of life inequality in Ethiopia was 27.6 years in 2011.

**Conclusion:**

Longevity has increased and the distribution of health in Ethiopia is more equal in 2011 than 2000, with length of life inequality reduced for all population groups. Still there is considerable potential for further improvement. In the Ethiopian context with a poor and highly rural population, inequality in length of life within wealth quintiles is considerably larger than between them. This suggests that other factors than wealth substantially contribute to total health inequality in Ethiopia and that identification and quantification of these factors will be important for identifying proper measures to further reduce length of life inequality.

## Introduction

The need to measure and document health inequality is well established [1-3], but exactly what to measure and how to do it have not been fully agreed see i.e. [4-7]. Most studies measure average health, as life expectancy and under-five mortality, and compare outcomes among pre-defined groups [7]. Measuring such between-group inequalities based on differences of means does not provide sufficient information about the individual distribution of health [8]. Describing within-group inequalities in health can therefore provide important information about population health to various stakeholders, like policy makers and public health researchers [9,10].

In this study, we looked at length of life inequality in different population groups in Ethiopia. Measuring length of life inequality is one among several ways of capturing overall health inequality. This was first done by Julian Le Grand [11], and in their 2009 paper Smits and Monden [12] highlight four reasons why this is a feasible measure: 1) a long and healthy life is valued across most societies and cultures, making it useful for comparisons among them; 2) as income and wealth are instrumental for reaching more essential goals, such as a long life, variations in length of life provide insight into these background factors; 3) inequality in length of life is more directly linked to absolute deprivation than, for example, inequality in income; and 4) the information needed to estimate length of life inequality is already easily available. Various assessments of length of life have been done on large datasets consisting of hundreds of life tables from many countries [13-16], but to our knowledge, no studies measure inequality and life expectancy within and between groups in a single country. For a short overview of selected population and health indicators in Ethiopia and Sub-Saharan Africa please see Table 1.

**Table 1:** Overview (2011 data if not stated otherwise)

	**Ethiopia**	**Sub-Saharan Africa**
Total population (000)	84 734	853 931
Urban population (% of total)	17.0	36.5
Life Expectancy at birth (years)	59.2	54.2*
GNI per capita (constant 2000 US$)	229	571
Poverty headcount ratio at 2$ a day (% of population)	66.0	69.9
Health expenditure per capita (current USD)	16.6	95
Physicians per 1 000 population	0.02*	0.16*
Total fertility rate (births per woman)	4.0	4.9
Maternal mortality rate	350*	500*
Under-5 mortality rate	77	108

*2010 [35].

The report from the Commission on Social Determinants of Health in 2008 acknowledges that specific national and local contexts have to be taken into consideration in order to reduce health inequities [1], meaning that in an Ethiopian context, with high poverty rates and a poorly developed health system, more information about health distribution and its relation to social determinants of health is required. Ethiopia, the second most populated country in Africa, has achieved impressive and important improvements in population health in the last few decades. Average life expectancy at birth has increased from 44 years in 1990 to 54 years in 2009 [17], and similar achievements have been shown in a variety of indicators related to health and development [18,19], reflecting the efforts made by the government in addressing these challenges. Still, allocation of health resources will continue to be a difficult task. Knowledge about the distribution of health in Ethiopia can also be of interest to other Sub-Saharan countries as there is a general lack of such data from the region.

We aim to model life expectancy and length of life inequality across gender, urban–rural residence and a national total for the years 2000 and 2011, and for wealth quintiles for 2011. Then we compare these within- and between group inequalities. As no good quality vital registration data exists, we believe that modeling health distribution using available summary measures will be of great value. Estimates of life expectancy and length of life inequality between and within population groups will provide a novel understanding of health distribution in Ethiopia, and it could serve as an important baseline for both theoretical work and for concrete policy making and priority setting.

## Methods

We modeled life expectancy and length of life inequality for Ethiopian population groups using a model life table system. We used available under-five and adult mortality rates as input to generate population-group specific life tables with estimates of life expectancy and age-specific mortality for different age-groups. This is used to estimate length of life inequality both within and between groups, calculated as Gini health scores (Gini_H_), absolute length of life inequality (ALI), concentration indexes (CI) and absolute differences.

### Life table modeling

To produce group-specific abridged life tables we used the MODMATCH modified logit life table system (MODMATCH) [20]. This method is used by WHO in areas with poor vital registration, including the World mortality in 2000-publication [21], and a similar method is used in the Global Burden of Disease Study 2010 [22]. MODMATCH builds on the Brass Logit life table system [23] which relies on an observed structural relationship between survival curves in life tables. With a linear relationship between the logit of their survivorship probabilities, these curves can be related to each other [24]. By using mathematical transformation based on two parameters, new unique life tables can be produced from this single standard life table. In MODMATCH, this transformation has been modified to better tackle the systematic errors observed in the original system [24].

To model life expectancy for groups, the modified logit life table system requires both under-five and adult mortality input. While under-five mortality data by gender, urban–rural residence and wealth quintiles are available from the 2011 EDHS [25], adult mortality stratified by the same groups are not [26]. This lack of basic knowledge concerning adult mortality is well-known [27]. We estimated adult mortality rates for urban and rural population and for the five wealth quintiles using weighted ratios of their respective under-five mortality rates, adjusted with a factor *x* (see appendix for further explanation).

### Measuring inequality

We used the Gini index to measure inequality in length of life. Originally developed for measuring economic inequality [28], it can also successfully be used to measure inequalities in health [11,29].

We calculate the Gini health index (Gini_H_) using Wagstaff’s formula

$${\text{Gini}}_{H}=1-\frac{v\sum_{i=1}^{T}f_{t}h_{t}{\left(1-R_{t}\right)}^{\left(v-1\right)}}{\mu }$$

where *f*_*t*_ is the sample proportion in the *t*th group, *h*_*t*_ is the length of life for the *t*th group, *R*_*t*_ its fractional rank, and μ is the average length of life for the total population which corresponds to average life expectancy at birth [29]. The parameter *v* reflects the aversion to inequality and is set to *v* = *2* as in the standard Gini [30]. The Gini_H_ calculates the *distribution* of inequality based on the number of persons dying in each age-group ranked from early to late death, extracted from the life tables produced in MODMATCH. The coefficient range from 0 to 1, where 0 describes perfect equality and 1 describes perfect inequality. Applied to length of life inequality, a Gini_H_ score of 0 implies that everyone dies at the same age, while a score of 1 corresponds to one person having a long life while all others die at birth.

Smits and Monden have calculated relative length of life inequality (RLI) based on the Gini_H_ index [12]. To simplify interpretation and estimate the within-group inequality in length of life we introduce a new measure, absolute length of life inequality, in addition to the Gini_H_ coefficient. The ALI should be interpreted as the average deviation in life expectancy between two randomly picked individuals in a population and is calculated as

$$\text{ALI}=\text{LE}\times {\text{Gini}}_{H}\times 2$$

with *LE* being the life expectancy in the population and *Gini*_*H*_ the Gini_H_ coefficient describing the distribution of age-specific deaths. This should provide a more intuitive interpretation of the inequality, as a lower absolute length of life inequality signifies more equal longevity for the population. LE multiplied with Gini_H_ gives one person’s expected deviation from the mean, but by multiplying it with two it describes the difference between two person’s estimated LE: an ALI of 20 years should be interpreted as if two individuals are selected at random, their estimated life expectancy would differ by 20 years. In this way, the ALI can easily be compared with measures of inequality that use differences between average groups, as the method of calculating differences in LE between population groups.

To calculate between-group inequalities, we used estimates of life expectancy to calculate concentration indices and absolute differences between the groups. The concentration index (CI) derives from the concentration curve, where individuals are ranked according to their relative socioeconomic position on the x-axis and with the y-axis presenting the cumulative proportion of health in these individuals. When this curve is plotted, its deviation from the diagonal Line of Equality (LoE) can be estimated, and the CI is defined as twice the area between the curve and the line of equality [31]. The CI ranges from −1 to 1, where a positive CI represents a concentration of a wanted health variable (like life years) among the best off in society. The absolute difference between wealth quintiles was calculated as the difference in life expectancy between the highest and lowest wealth quintile.

### Data selection

We used under-five mortality data from the 2011 and 2000 Ethiopia Demographic and Health Survey (EDHS) [25,32]. The EDHS is part of the larger MEASURE DHS project funded by USAID, in which countries conduct national household surveys to monitor and evaluate a range of indicators related to population, health and nutrition. In the 2011 EDHS, a total of 17 817 households (31% urban, 69% rural) were selected, with a household response rate of 98.1% and with 16 515 women and 14 110 men aged 15–49 interviewed [25]. For the 2000 EDHS, 14 642 households (26% urban, 74% rural) were selected, with a response rate of 99.3% and with 15 367 women and 2 607 men interviewed [32]. Under-five mortality by wealth quintiles was not available in the EDHS 2000, and therefore only gender and residence groups were compared across time.

Wealth quintiles are used as proxies of socioeconomic position. They are measured in a standardized way in each DHS by an asset index. The index is constructed from household asset data and dwelling characteristics such as ownership of a television, a bicycle or car, source of drinking water, sanitation facilities and type of material used for flooring [33]. Based on the asset index, the population is divided into five quintiles. These wealth quintiles are commonly used as a method of estimating socioeconomic position, especially in low income countries where lack of data is customary.

Data used on adult mortality and life expectancy is from the Global Burden of Disease study 2010 (GBD 2010) [26]. The GBD 2010 is a large international collaboration aiming to provide strong evidence-based assessment of health problems worldwide, led by the Institute of Health Metrics and Evaluation at the University of Washington, which in December 2012 published their findings in a special edition of The Lancet [34]. The urban–rural and male–female ratio are from the World Development Index [35].

## Results

**Figure 1:**
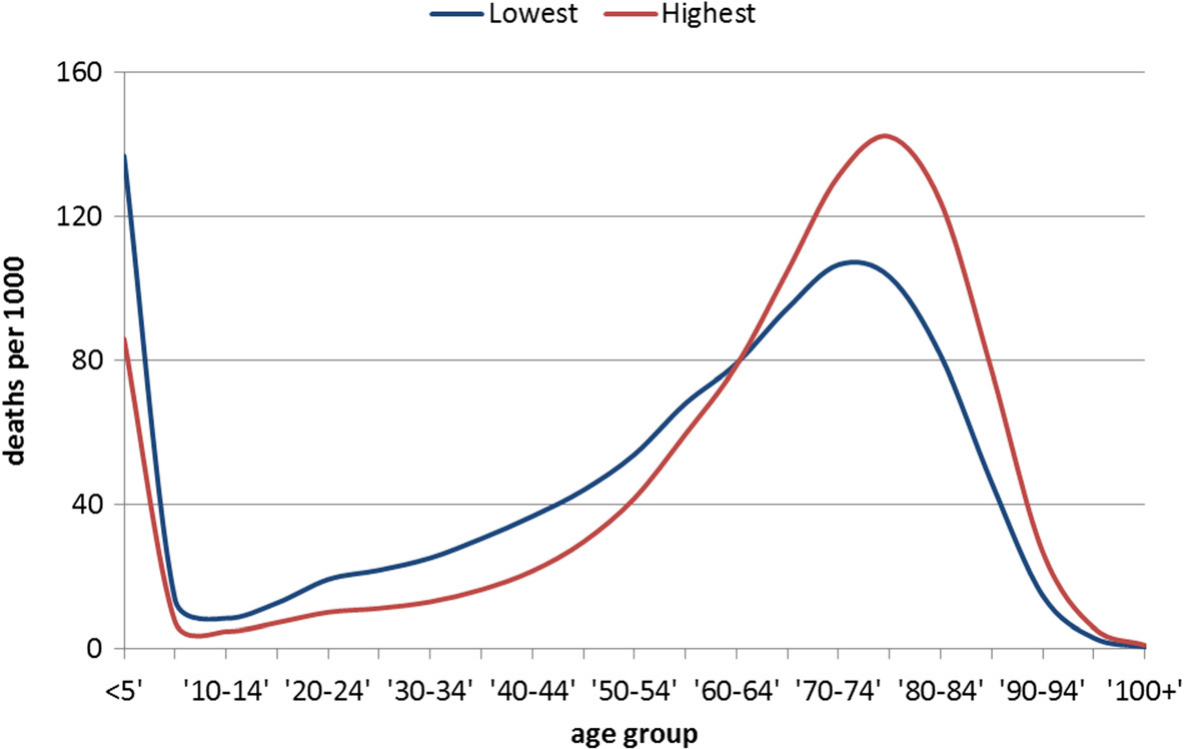
**Mortality distribution for highest and lowest wealth quintile 2011.** Mortality given as deaths per 1000 (y-axis) plotted against five-year age groups (x-axis).

Figure 1 show the distribution of deaths by five year age-groups for the highest and lowest wealth quintile in Ethiopia. This illustrates the inequality in length of life within the two groups. From this mortality distribution, we have calculated within-group length of life inequality which we present in Table 2 together with between-group inequality for the wealth quintiles.

**Table 2:** Length of life inequality estimates within and between wealth quintiles in Ethiopia, 2011

		***Within*-*group inequality***		***Between*-*group inequality***	
	**LE**	**Gini**_**H**_	**ALI (years)**	**CI**	**Abs.diff (years)**
	**(years)**				
**Wealth quintile**					
Lowest	53.4	0.29	30.6		
Second	56.2	0.26	29.4		
Middle	60.6	0.22	27.0	0.030	9.0*
Fourth	59.9	0.23	27.5		
Highest	62.5	0.21	25.9		

* calculated between highest and lowest wealth quintile.

We see a clear socioeconomic gradient in Ethiopia, with a life expectancy ranging from 53.4 years in the lowest wealth quintile to 62.5 years in the highest quintile - an absolute difference of 9 years. There is also a correspondingly decrease in length of life inequality from the lowest to the highest quintile: a Gini_H_ score of 0.29 in the lowest wealth quintile and 0.21 in the highest quintile.

**Figure 2:**
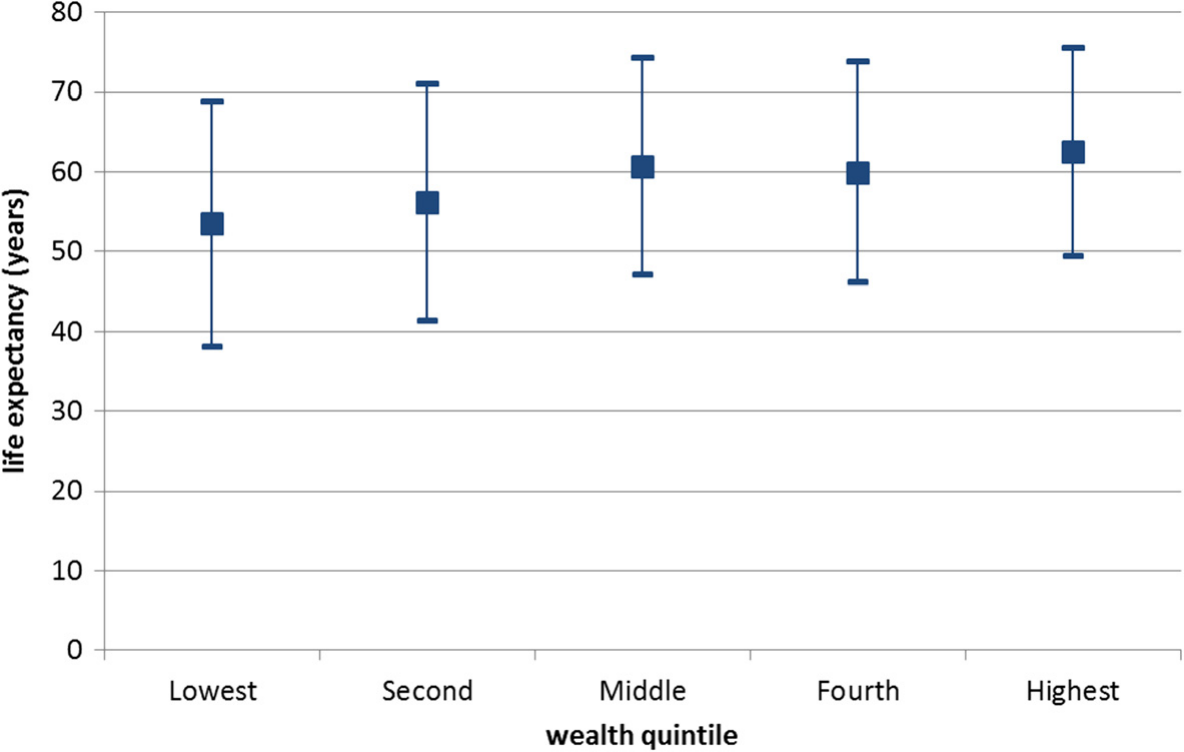
**Within-group and between-group inequality among wealth quintiles 2011.** Life expectancy (central dots) and absolute length of life inequality (high and low bar) for wealth quintiles indicates larger within- than between-group inequality.

The absolute difference in life expectancy between the highest and lowest quintile was 9 years, and the CI was estimated to 0.030. As we can see in Table 2, the absolute length of life inequality within the various wealth quintiles varied from 25.9 years to 30.6 years. This is plotted in Figure 2 as estimated life expectancy ± ALI/2 and shows that length of life inequality is far greater *within* quintiles than *between* them. We also see that the 9 year difference was relatively small compared with the 27.6 year total absolute length of life inequality in Ethiopia.

In Table 3, we present estimates of length of life inequality across gender, urban–rural residence, and for the total population in 2000 and 2011, measured as both within-group and between-group inequality.

**Table 3:** Length of life inequality and life expectancy estimates for population groups in Ethiopia, 2000 and 2011

			***Within-group inequality***		***Between-group inequality***	
		**LE**	**Gini**_**H**_	**ALI (years)**	**CI**	**Abs.diff (years)**
		**(years)**				
**Gender**						
2000	Male	48.9	0.33	32.6	0.005	1.7
	Female	50.6	0.33	33.4		
2011	Male	56.7	0.25	28.6	0.014	3.2
	Female	59.9	0.23	28.0		
**Residence**						
2000	Urban	55.1	0.28	30.9	0.008	6.2
	Rural	48.9	0.34	33.3		
2011	Urban	63.1	0.20	25.5	0.013	5.6
	Rural	57.5	0.25	28.8		
**National**						
2000	Total	49.7	0.33	33.1	-	-
2011	Total	60.9	0.23	27.6	-	-

Life expectancy has increased and within-group length of life inequality has decreased for all groups between 2000 and 2011, and there are greater length of life inequality among males and rural residents compared to females and urban residents. In 2011 males and females had a Gini_H_ score of 0.25 and 0.23 respectively, decreasing from 0.33 in 2000. In terms of absolute length of life inequality, males had 28.6 and females 28.0 years in 2011, compared to 32.6 and 33.4 years in 2000. The absolute difference in life expectancy between males and females was 3.2 years in 2011 and 1.7 years in 2000, corresponding to a CI of 0.014 and 0.005 respectively.

There is greater length of life inequality among rural than urban residents, with Gini_H_ scores of 0.25 and 0.20 in 2011, compared to 0.34 and 0.28 in 2000. Absolute length of life inequality has been reduced from 33.3 and 30.9 years for rural and urban residents in 2000, to 28.8 and 25.5 years in 2011. The absolute difference in life expectancy was 5.6 years in 2011 and 6.2 years in 2000, with CI of 0.013 and 0.008. The total length of life inequality in Ethiopia has also decreased; from a Gini_H_ score of 0.33 in 2000 to 0.23 in 2011. In the same period, life expectancy has increased from 49.7 years to 60.9 years.

## Discussion

Our findings show that length of life inequality has decreased and life expectancy increased in Ethiopia from 2000 to 2011 and that within-group inequality are substantially larger than between-group inequality. Inequality between wealth quintiles only account for about one third of total health inequality. We find larger length of life inequality between males, rural residents, and the less wealthy, compared to women, urban residents and the wealthier. Estimates of life expectancy follow the same pattern. By estimating length of life inequality and life expectancy for females and males, urban and rural residents, and for wealth quintiles, we offer a new and more comprehensive picture of population level health in Ethiopia. This is important, as it can provide a baseline for priority setting and resource allocation in Ethiopia.

There are some limitations to our findings. Length of life inequality does not fully capture the overall health inequality in a population, and we do not claim that it should be the only indicator used to describe health. We do think it provides important and supplementary information to other well-known measures, like life expectancy, DALYs, and mortality rates, as it describes the *distribution* of health. A weakness in the MODMATCH life table system is that it can underestimate mortality at younger ages and overestimate mortality at older ages in countries with high prevalence of HIV/AIDS [22]. However, HIV prevalence in Ethiopia at 1.5 is relatively low compared to other Sub-Saharan countries [35], making this less a problem for our findings. The lack of adult mortality data makes it necessary to use estimates. We believe our estimates based on weighted under-five mortality rates are reasonable as many of the associative factors are the same, and our results are comparable to analysis done in other countries [36,37].

The wealth index is only a proxy of socioeconomic position, and although it is commonly used, it does not capture the full impact of other socioeconomic determinants like income and education. Measuring only the differences between the highest and lowest group obviously neglects the middle groups. Still, absolute differences in health between groups are among the most commonly used measures of health inequality between socioeconomic groups and we therefore think its use is justified. By comparing it to ALI, an individual measure of inequality, we want to illustrate the need for individual health measures as a supplement to the average measures between population groups.

Traditionally, between-group inequalities have received more attention from researchers, in addition to claims that differences between pre-defined socioeconomic groups are what we should be morally concerned with [38]. In Figure 2 we illustrate that within-group inequalities are considerably larger than the between-group inequality. The within-group inequalities are about three times larger than the absolute difference between the highest and lowest wealth quintile, questioning to what extent between-group comparisons actually capture what we expect it to do.

From our findings we can also see that wealth only gives a limited contribution to total health inequality. This is indicated by comparing the total inequality in 2011 of 27.6 years with the absolute difference between the wealth quintiles: if we randomly select two individuals, one from the highest and one from the lowest wealth quintile, their average difference in life expectancy would equal the absolute difference in life expectancy between the highest and lowest quintile of 9 years. If we then randomly select two individuals from the whole population, their expected difference in life expectancy would be 27.6 years. A full decomposition of factors associated with inequality in age at death could reveal how much of total inequality can be explained, and this calls for further analysis.

These findings demonstrate how wealth alone provides an insufficient explanation of health inequalities in Ethiopia. Wagstaff and van Doorslaer have estimated socioeconomic inequality to be about 25% of total inequality [39], and this concur with our findings. Tuljapurkar [40] and Edwards and Tuljapurkar [14] have similar findings, with education and household incomes having a greater impact on averages and less effect on inequality itself. Tuljapurkar claims that his results shows that ‘…reducing some kinds of socioeconomic inequality will have little or no effect on inequality in age at death’ [40].

Both the general wealth level and the method of assessing wealth in Ethiopia can partly explain our findings. According to the World Bank, in 2005 77.6% of the population lived on less than 2 USD per day [41]. This implies that almost everyone in the four lowest wealth quintiles is extremely poor. Therefore, with such a low general level, one may not expect to observe great differences in health outcomes between these groups. There are also concerns that the DHS wealth index in general have an urban bias and that it fails to separate the extremely poor from the poor [42]. Both these concerns may therefore apply to Ethiopia, with its vast share of poverty and highly rural population.

As in most low income countries, Ethiopia has a rural–urban migration pattern, with an increase of the urban population ratio from 14.7% to 17.0% from 2000 to 2011. This corresponds to an absolute growth in urban population ratio of 2.3%. For the Sub-Saharan region as a whole, the absolute growth in urban population ratio was 4.5%, as the urban population ratio increased from 32.0% to 36.5% [35]. This means that Ethiopia has a significant lower share of urban populated people than its regional average and that the urbanization rate is quite slow, at least compared to its region. We believe this makes our comparison of the inequality between urban and rural groups across time valid. Further, the Gini index is population insensitive [43], which mean that calculations of within-group inequality also can be compared across time even if the size of the groups changes.

The positive development from 2000 to 2011, with a decrease in length of life inequality and an increase in life expectancy, can be seen as a part of the positive general development in Ethiopia. Efforts like the Health Sector Development Program [18] and the introduction of the health extension workers have led to important health improvements. A continuous focus will be required, including work to increase health spending. The Ethiopian government spent 19 USD per capita on health in 2008 [17], which according to a WHO task force is 41 USD short of the 60 USD recommended to spend in order to achieve the health Millennium Development Goals [44].

Our findings suggest that other factors than wealth contributes to length of life inequality in Ethiopia. We do not claim that an unequal distribution of wealth is acceptable, but we ask if health inequalities in Ethiopia can be reduced by also addressing other factors. With coverage rates for many important interventions still being low [25] it is reasonable to believe that inequality in access to health services also contribute to inequality. Other health determinants, like infrastructure, quality of care, and coverage of health workers also contribute in various amounts, and may well be quantitatively more important than socioeconomic factors. We also claim that today’s measures of health inequality do not capture the individual distribution of health and propose absolute length of life inequality as a measure to describe individual inequality. If *distribution* of health is to be included as an important part of a summary measure population health, as we believe is should, more work is needed both to identify and quantify contributing factors.

## Conclusion

Our findings support the observed positive trend in Ethiopian population health: life expectancy has increased and the distribution of health is more equal, with length of life inequality reduced for all population groups. Still there is a large potential for further improvement. In the Ethiopian context, with a poor and rural population, inequality in length of life within wealth quintiles is considerably larger than between them, implying that factors other than wealth make a substantial contribution to total health inequality. If this unequal distribution of health is of concern, measures must be taken to reduce inequality, including further work to identify and quantify contributing factors.

## Appendix

A thorough description of the MODMATCH modified logit life table system is available in Murray et al’s 2003 paper [24]. We have estimated adult mortality rates for urban–rural residents and for five wealth quintiles using weighted under-five mortality ratios, adjusted with a factor *x*. As an example was the female adult mortality rate for rural residents calculated as

$$F{45q15}_{\text{RUR}}=F{45q15}_{\text{TOT}}\times {U5}_{\text{RUR}}\times p_{\text{RUR}}\times X$$

where *F45q15*_*TOT*_ is the gender-specific adult mortality rate, *U5*_*RUR*_ the ratio of rural to urban under-five mortality rate, *p*_*RUR*_ the rural population share in Ethiopia and *x*,identified by validating our estimated mortality rates with the rates given by the GBD 2010. This was done as we wanted to use the tendency and not the exact mortality pattern as for the under-five rates. This adjustment can be written as

$$x=\frac{1}{\sum_{i=1}^{n}\left({U5}_{i}\times p_{i}\right)}$$

with *U5*_*i*_ the ratio of group *i*'s under-five mortality rate, and *p*_*i*_ the population share for group *i* for *n* different groups from *n*_*1*_ to *n*_*i*_. Summarizing the estimate for female adult mortality in rural areas in 2010 give

$$\begin{array}{l} F{45q15}_{\text{RUR}}=0.2739\times 1.373\times 0.832\times 0.763\\ \;\;\;\;=0.287 \end{array}$$

A unique x was calculated for wealth quintiles in 2010 and urban–rural residence in 2010 and 2000, and group specific adult mortality rates was estimated. There was no need to adjust input when modeling life tables based on gender, as specific under-five and adult mortality data was available. Under-five mortality by wealth quintiles were not available in the EDHS 2000, and therefore are only gender and residence groups compared over time. For urban–rural and wealth groups, male and female age-specific mortality rates was summarized using gender ratios from the World Bank Database [41].

## Abbreviations

0q5: Under-five mortality rate probability of dying between 0 and 5 years; 45q15: Adult mortality rate probability of dying between 15 and 60 years; ALI: Absolute length of life inequality; CI: Concentration index; DALY: Disability-adjusted life year; DHS: Demographic and health survey; EDHS: Ethiopia demographic and health survey; GBD: 2010 global burden of disease study 2010; LE: Life expectancy; RLI: Relative length of life inequality; WHO: World health organization; USD: United States dollar.

## Competing interests

The authors declare that they have no competing interest.

## Authors’ contributions

EJT and OFN designed the study, preformed the analysis, interpreted the result and wrote the paper. MA assisted in data analysis, interpretation of the data and writing of the paper. All authors read and approved the final manuscript.
